# Evaluating the Corrected Femoroepiphyseal Acetabular Roof Index in Patients With Borderline Dysplasia Undergoing Periacetabular Osteotomy or Hip Arthroscopy

**DOI:** 10.1177/23259671241307648

**Published:** 2025-01-30

**Authors:** Dimitris Dimitriou, Georgios Neopoulos, Lukas Jud, Patrick O. Zingg

**Affiliations:** † Balgrist University Hospital, Orthopaedic Department, University of Zurich, Zurich, Switzerland; Investigation performed at Balgrist University Hospital, Orthopaedic Department, Zurich, Switzerland

**Keywords:** borderline hip dysplasia, femoroepiphyseal acetabular roof index, hip arthroscopy, hip instability, hip preservation, osteoarthritis, periacetabular osteotomy

## Abstract

**Background:**

Identifying hip instability in symptomatic patients with borderline dysplasia of the hip (BDH) is of paramount importance, as it can influence both surgical decision-making and surgical outcomes. The femoroepiphyseal acetabular roof (FEAR) index is strongly affected by the hip adduction/abduction angle during the pelvic radiograph, which has not yet been considered in the recommended threshold values.

**Purpose:**

To compare the corrected FEAR index in symptomatic patients with BDH treated with pelvic periacetabular osteotomy (PAO) or hip arthroscopy.

**Study Design:**

Cohort study; Level of evidence, 3.

**Methods:**

Patients with symptomatic hips and radiographical BDH were categorized into 2 cohorts. The first cohort included patients treated with PAO (n = 42) and the second cohort consisted of patients treated with hip arthroscopy due to symptomatic femoroacetabular impingement (n = 50). All patients presented with good patient-reported outcomes at the final follow-up. The FEAR index was measured on the pelvic radiograph at the initial hip adduction/abduction angle (uncorrected FEAR index) and after correcting the hip abduction angle to 0° (corrected FEAR index). Negative values of the FEAR index represent a lateral closing angle, whereas positive values represent a lateral opening angle. As for the hip adduction/abduction angle, negative values represent adduction, whereas positive values represent abduction.

**Results:**

The corrected FEAR index varied significantly from the uncorrected FEAR index in both groups with a mean difference of 6°± 4° in patients treated with PAO and 5°± 5° in patients treated with hip arthroscopy. The corrected FEAR index in patients with BDH treated with hip arthroscopy (−11°± 8°) was significantly lower (*P* < .001) compared with the patients with BDH treated with PAO (−7°± 7°) (Table 1). The optimal threshold for the corrected FEAR index was −13° (odds ratio, 7.8 [95% CI, 2.6-23.1]; *P* < .001), which yielded a sensitivity of 85% and a specificity of 52%, distinguishing the 2 surgical cohorts.

**Conclusion:**

The corrected FEAR index might vary significantly from the uncorrected FEAR index, which is highly dependent on the hip adduction/abduction angle during the pelvic radiograph. Symptomatic patients with BDH treated with PAO exhibit a corrected FEAR index of ≥–13° compared with those with BDH treated with hip arthroscopy for impingement symptomatology.

Borderline dysplasia of the hip (BDH) is defined as a lateral center-edge angle (LCEA) of 18° to 24°^4,17^ and has a prevalence of 19.8% to 23.3% in the asymptomatic general population and 12.8% in symptomatic patients.^6^ Despite its high prevalence, the optimal treatment option in patients with symptomatic BDH remains controversial.^14^ Depending on the type of symptoms, either instability or impingement, the optimal treatment of BDH may vary. Periacetabular osteotomy (PAO) is preferred in patients with predominately instability symptoms, as it addresses the structural deformity at the expense of greater surgical exposure,^7,10^ prolonged recovery,^8^ and higher potential complications.^3^ However, an arthroscopic procedure is favored in patients with mainly femoroacetabular impingement (FAI) symptoms, which addresses the intra-articular pathology (eg, labral tear or associated cam deformity) in a minimally invasive approach but without correcting the acetabular bony pathology.^5^ It is reported that the most common cause of arthroscopic failure—defined as conversion to total hip arthroplasty or pelvic/femoral osteotomy, a modified Harris Hip Score (mHHS) of ≤70, and complications—was inappropriate patient selection (ie, misdiagnosed as stable hips).^11^ Therefore, an accurate diagnosis of hip instability in treating patients with BDH is of paramount importance.

The femoroepiphyseal acetabular roof (FEAR) index, defined as the angle formed between a line extending from the femoral head physeal scar and the sclerosis of the acetabular sourcil, was introduced in 2017 by Wyatt et al^20^ as a radiographic measure to differentiate between patients with BDH and true instability as opposed to FAI. In their original publication, hips with a FEAR index of <5° were considered stable. Positive values of the FEAR index indicate a lateral opening angle, while negative values indicate a lateral closing angle. Several studies reported varied optimal thresholds of the FEAR index to identify hip instability ranging from^16^–1.3° to^2,13^ 3° in BDH and −5° in nondysplastic hips,^18^ with high specificity (up to 92.4%) and accuracy (up to 69.4%). However, a recent external validation of the FEAR index^15^ demonstrated a very low sensitivity in detecting the clinical diagnosis of instability (33% for the threshold of 5° and 39% for a threshold of 2°), whereas a threshold of −5° was required to reach an adequate sensitivity of 74%.

The FEAR index is highly dependent on hip abduction during the pelvic radiograph, where for a simple and single directional sense, positive values represent hip abduction and negative values represent hip adduction. Even though conventional anteroposterior pelvic radiographs are routinely performed in a standardized manner with the hip in the neutral position, a recent study^9^ found that the mean hip abduction during pelvic radiographs was −5.3°± 3.1° (range, –14° to 2°), which affected the FEAR index, demonstrating a mean difference from the neutral position (abduction/adduction: 0°) of 9.2°± 7° (range, –27° to 10°). Therefore, the present study aimed to (1) compare the corrected FEAR index in patients with BDH who had mainly hip instability symptoms and were treated with PAO and patients with BDH who had predominantly FAI symptoms and were treated with hip arthroscopy and (2) report the optimal threshold of the corrected FEAR index, distinguishing these 2 cohorts of patients with BDH.

## Methods

### Inclusion and Exclusion Criteria and Study Design

The study was approved by our institution’s internal review board and the ethical committee (Zurich Cantonal Ethics Commission, BASEC-Nr. 2022-00950). The study was conducted entirely at the authors’ institution. The medical records and hip radiographs of all patients aged 16 to 40 years who presented in our outpatient hip clinic between January 2005 and December 2020 were retrospectively reviewed. The institutional outpatient database and operative records were searched for the following diagnoses: “hip dysplasia,”“borderline hip dysplasia,” or “LCEA 18° to 24°.” Patients with an LCEA between 18° and 24° and an adequate supine anteroposterior pelvic radiograph were included and categorized into 2 groups depending on their treatment method. Additional inclusion criteria for the first group—including patients with BDH and mainly symptoms of hip instability who underwent PAO—were improvements in their patient-reported outcomes—including mHHS, subjective hip value, Western Ontario and McMaster Universities (WOMAC) Osteoarthritis Index—at a minimum 5-year follow-up. Additional inclusion criteria for the second group, which consisted of patients with BDH and FAI symptoms treated with hip arthroscopy were improvements in their patient-reported outcomes (mHHS, WOMAC Osteoarthritis Index) at a minimum 1-year follow-up according to our clinic standards. The exclusion criteria were patients aged <16 years or >40 years at the time of the first consultation, patients without an adequate supine anteroposterior pelvic radiograph, patients with incomplete data collection, patients without an adequate minimum follow-up time of 5 years for the first group and 1 year for the second group, and hips who underwent a concomitant femoral osteotomy, previous surgery in the affected hip, post-Perthes, or previous epiphysiolysis femoris capitis. The criteria for an adequate anteroposterior pelvic radiograph were that lower limbs were internally rotated 15° to 25° from the hip, the entirety of the bony pelvis was imaged from the superior of the iliac crest to the proximal shaft of the femur, both obturator foramina, and acetabular teardrops appeared symmetrical, iliac wings had an equal concavity, greater trochanters of the proximal femur were visible in profile, the lesser trochanters were partially superimposed over the femoral neck, and that the sacrococcygeal joint was 1 to 3 cm superior to the upper surface of the pubic symphysis.

### Patient Groups

Patients were categorized into 1 of 2 groups based on the surgical treatment.

#### BDH Patients Treated With PAO

Patients with BDH and symptoms of hip instability—defined as (1) having a history of groin pain during weightbearing activities (running, stairs) and (2) experiencing hip instability reproduced with hip apprehension test in the physical examination—were selected from a consecutive retrospective cohort comprising patients with BDH who underwent PAO between January 2009 and January 2016 with correction of the LCEA.

#### BDH Treated With Hip Arthroscopy

The group consisted of patients with BDH and predominantly FAI symptoms (groin pain with hip flexion activities, positive anterior impingement test, and concomitant cam deformity radiographically) who underwent hip arthroscopy with labral repair and correction of their cam deformity.

### Radiographic Measurements

**Figure 1.**
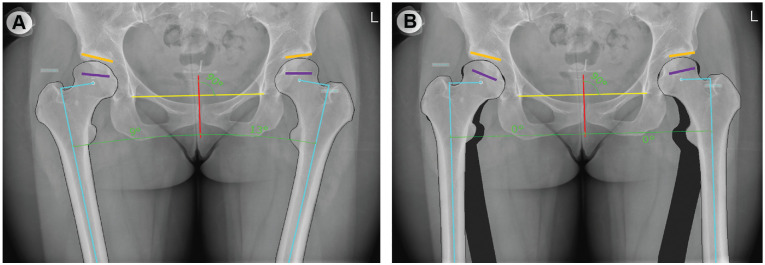
A pelvic anteroposterior radiograph demonstrating (A) the initial position of the hips as taken in the radiology department and (B) after correction of the hip adduction/abduction angle. The hip adduction/abduction angle was defined as the angle between a vertical line (red line) to the tangent to the pelvic teardrops (yellow line) and the femoral shaft axis (light blue line). The acquired radiograph demonstrated 13° of adduction on the left side and 9° of adduction on the right side. The FEAR index was defined as the angle between the physeal scar (purple line) and the sclerosis of the acetabular sourcil (orange line). The uncorrected FEAR index demonstrated in (A) is positive (lateral opening angle), whereas the corrected FEAR index, demonstrated in (B) is negative (lateral closing angle). FEAR, femoroepiphyseal acetabular roof.

The pelvic radiographs of the initial presentation at the clinic were imported into the surgical planning software mediCAD (Hectec GmbH, Version 7.0; Altdorf) for further analysis. The hip abduction angle was defined as the angle between a vertical line to the tangent of the pelvic teardrops and the femoral shaft axis (Figure 1). The FEAR index (uncorrected) was measured between a line on the physeal scar of the femoral head and a tangent to the sclerosis of the acetabular sourcil (Figure 1A). To measure the corrected FEAR index, the hip rotation center was determined by the center of the femoral head; the proximal femur was cut out and the hip abduction was corrected to neutral (ie, 0°) using the cut-out function of the mediCAD software (Figure 1B), as previously described.^9^ For the FEAR index, lateral opening angles were handled as positive values, whereas lateral closing angles were handled as negative values. As for the hip abduction angle, negative values represent adduction, whereas positive values represent abduction.

### Statistical Analysis

Means, standard deviations, and ranges were used to present the data. The 2-way paired *t* test was used to compare the uncorrected and corrected FEAR index within the groups, whereas the unpaired *t* test was used to identify potential significant differences in the FEAR index between groups. A receiver operating characteristic (ROC) curve analysis was performed to define the optimal threshold value of the corrected FEAR index for detecting hip instability. For this analysis, patients who underwent PAO were considered to have unstable hips, whereas patients treated with hip arthroscopy were considered stable. The Youden index^12^ was utilized to determine the ideal threshold value with the highest sensitivity and specificity. Based on the calculated threshold value, the odds ratio was calculated to determine the probability of a patient having an unstable hip according to the corrected FEAR index. The level of significance was set at *P*≤ .05. All the statistical analyses were performed using SPSS Version 23 software (SPSS Inc).

## Results

A total of 92 patients met the inclusion and exclusion criteria (Table 1).

**Table 1:** Descriptive Data, Follow-up, and Radiographical Measurements^*a*^

	Patients With BDH Treated With PAO (n = 42)	Patients With BDH Treated With Hip Arthroscopy (n = 50)
Age, y	27 ± 7 (16 to 39)	30 ± 8 (16 to 40)
Sex, female	33 (83)	29 (63)
Left side	16 (38)	20 (40)
BMI, kg/m^2^	23 ± 4 (18 to 30)	23 ± 3 (18 to 30)
Follow-up, y	6 ± 1 (5 to 8)	1.2 ± 0.2 (1 to 2)
LCEA, deg	21 ± 2 (18 to 24)	22 ± 2 (18 to 24)
Acetabular index, deg	12 ± 4 (1 to 21)	10 ± 5 (1 to 15)
Uncorrected FEAR Index, deg	−2 ± 7 (−16 to 16)	−6 ± 6 (−23 to 6)
Corrected FEAR Index, deg	−7 ± 7 (−23 to 10)	−11 ± 8 (−28 to 10)

a Data are presented as mean ± SD (range) or n (%). BDH, borderline dysplasia of the hip; BMI, body mass index; deg, degree; FAI, femoroacetabular impingement; FEAR index, femoroepiphyseal acetabular roof index; LCEA, lateral center-edge angle; PAO, periacetabular osteotomy.

### BDH Patients Treated With PAO

A total of 42 hips in 40 patients (female, n = 33; left hip, n = 16) were identified. The mean age at surgery (PAO) was 27 ± 7 years (range, 16 to 39 years) and the mean follow-up was 6 ± 1 years (range, 5 to 8 years). The mean body mass index (BMI) was 23 ± 4 kg/m^2^ (range, 18 to 30 kg/m^2^). The mean hip abduction angle was −5°± 3° (range, –16° to 16°). The mean uncorrected and corrected FEAR indexes were −2°± 7° (range, –16° to 16°) and −7°± 7° (range, –23° to 10°), respectively. The corrected FEAR index was significantly different from the uncorrected FEAR index with a mean difference of 6°± 4° (*P* < .001).

### BDH Treated With Hip Arthroscopy

A total of 50 hips in 46 patients (female, n = 29; left hip, n = 20) were identified. The mean age at surgery (hip arthroscopy) was 30 ± 8 years (range, 16 to 40 years) and the mean follow-up was 14 ± 1 months (range, 12 to 24 months). The mean BMI was 23 ± 3 kg/m^2^ (range, 18 to 30 kg/m^2^). The mean hip abduction angle was −5°± 4° (range, –11° to 5°). The mean uncorrected and corrected FEAR indexes were −6°± 6° (range, –23° to 6°) and −11°± 8° (range, –28° to 10°), respectively. The corrected FEAR index was significantly different from the uncorrected FEAR index with a mean difference of 5°± 5° (*P* < .001). The corrected FEAR index in patients with BDH treated with hip arthroscopy (−11°± 8°) was significantly lower (*P* < .001) compared with the patients with BDH treated with PAO (−7°± 7°) (Table 1).

### ROC Curve Analysis and Odds Ratio

**Figure 2.**
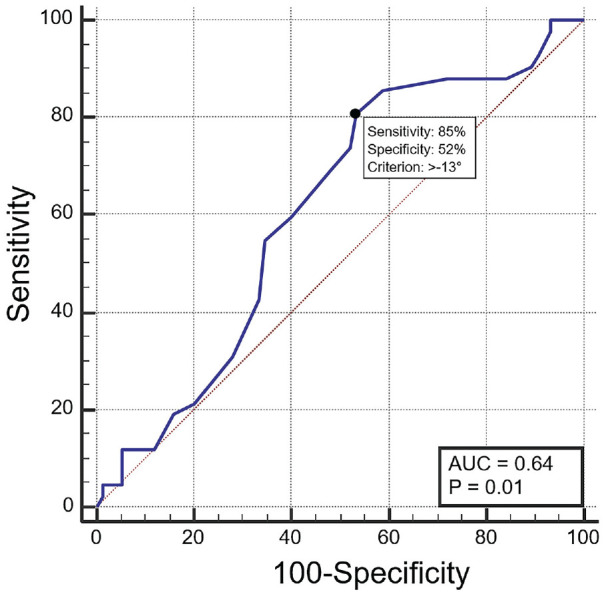
Summary of the ROC curve analysis, performed to define the optimal threshold of the corrected FEAR index in distinguishing between patients in the 2 cohorts. The Young index (black circle) demonstrated that the optimal threshold of the corrected FEAR index was −13°, which yielded a sensitivity of 85% and a specificity of 52%. AUC, area under the curve; FEAR, femoroepiphyseal acetabular roof; ROC, receiver operating characteristic.

The ROC curve analysis demonstrated that the optimal threshold for the corrected FEAR index was −13°, which yielded a sensitivity of 85% and a specificity of 52% in distinguishing between patients with BDH treated either with PAO for instability symptoms of hip arthroscopy for FAI symptoms (area under the curve, 64%) (Figure 2). The positive and negative predictive values were 52% and 84%, respectively, while the accuracy of the corrected FEAR index in distinguishing between patients in the 2 cohorts was 68%. Symptomatic patients with BDH with a corrected FEAR index of ≥–13 demonstrated a significantly higher probability of being treated with PAO (odds ratio, 7.8 [95% CI, 2.6 to 23.1]; *P* < .001) compared with patients with a FEAR index of <–13°.

## Discussion

The most important finding of the present study was that the corrected FEAR index was significantly different from the uncorrected FEAR index. The optimal threshold of the corrected FEAR index in distinguishing between patients with BDH treated successfully in terms of clinical outcomes—either with PAO or hip arthroscopy—was −13°.

Hip instability in the setting of BDH is challenging but very relevant for treatment outcomes. Patients with an unstable hip treated with hip arthroscopy might suffer from persistent pain due to instability, arthritis progression of the joint, and worse clinical outcomes (up to 50% of the patients in some studies).^1,11,21^ Zimmerer et al,^22^ in a retrospective study of 36 patients with BDH who underwent arthroscopic treatment of a concomitant FAI, reported poor outcomes in patients with a FEAR index of <2° and posterior wall deficiency but also in patients with a FEAR index of >2° and anterior wall deficiency. Similarly, Wong et al,^19^ in a retrospective analysis of 140 patients with BDH, found no difference in clinical outcomes in patients with a FEAR index of <2° compared with patients with a FEAR index of >2° after hip arthroscopy for FAI. The above-mentioned studies implied that the FEAR index in isolation may not be adequate to detect poor clinical outcomes.

The diagnostic accuracy of the FEAR index, specifically its sensitivity and specificity, varies in the literature and different threshold values have been suggested.^2,13,16,18^ An optimal screening test should have high sensitivity and identify most patients with the finding of interest, whereas a confirmatory test should have a high specificity. In the original report of Wyatt et al,^20^ which assessed the FEAR index in a single-surgeon cohort of 39 patients, a 5° threshold of the FEAR index was reported to yield a sensitivity and specificity of 78% and 80%, respectively. However, patients with classic acetabular dysplasia and BDH were included, which may have affected FEAR index performance. A subsequent study by the same group reported that a FEAR index of <2° demonstrated a sensitivity of 90% in detecting hip instability.^2^ In a recent external validation of the FEAR index in patients with BDH, Schwabe et al^15^ reported a poor sensitivity at both 5° and 2° thresholds (33% and 39%), whereas a threshold of −5° was required to achieve a sensitivity of 74% in identifying patients with a hip instability (based primarily on clinical decision and treatment, meaning hip arthroscopy for stable hips and PAO for unstable hips). Nevertheless, none of the studies that validated the utility of the FEAR index took into consideration the hip abduction angle during the pelvic radiograph, which significantly affects the FEAR index.^9^ This might be a reason for the inconsistent thresholds reported in the literature.

In the present study, the uncorrected FEAR index varied significantly from the corrected FEAR index in both BDH groups. The optimal corrected FEAR index in distinguishing between patients with BDH treated either with PAO or hip arthroscopy was −13°, which yielded a sensitivity of 85%; this highlights its potential as a screening tool in patients with BDH considered for surgical treatment. The present study emphasizes the importance of the corrected FEAR index in evaluating patients with BDH and symptoms of either instability or FAI, given its significant dependency on the hip abduction angle during pelvic radiographs.

### Limitations

The present study should be interpreted in light of its potential limitations. The most obvious drawback was the retrospective study design. However, due to the standardized clinical and radiological follow-up protocol and the excellent documentation through the orthopaedic surgeons at our institution, all of the patient data needed were available for the present analysis. Another important limitation to consider is the execution of the anteroposterior pelvic radiograph, conducted with patients in the supine position. The abduction and adduction of the hip may be affected and could differ between the supine and standing positions, potentially impacting the outcomes regarding the difference between a standard anteroposterior radiograph of the pelvis and the corrected version. A third limitation is the definition of hip instability. In the present study, hip instability was defined based on the patient history, clinical examination, and also according to good functional outcomes after PAO. However, as hip instability is not clearly defined in the literature and since patients treated with PAO had predominately instability symptoms, whereas patients treated with hip arthroscopy reported mainly FAI symptoms that improved after surgical treatment, the authors believe that the current classification of hip instability is meaningful, as the aim of the study was to define a threshold of the corrected FEAR index that would help in surgical decision-making.

## Conclusion

The present study confirmed that the corrected FEAR index varied significantly from the uncorrected FEAR index, which is highly dependent on the hip abduction angle during the pelvic radiograph. Patients with BDH and a corrected FEAR index of ≥–13° might demonstrate excellent clinical outcomes after PAO, which could guide surgical decision-making.
